# Supplementary material for: Inhibitory Activity of 4-Benzylidene Oxazolones Derivatives of Cinnamic Acid on Human Acetylcholinesterase and Cognitive Improvements in a Mouse Model
Source: Molecules. 2023 Nov 2;28(21):7392. doi: 10.3390/molecules28217392 (PMC10649417; doi:10.3390/molecules28217392)
Supplement: Supplementary file 1 [file molecules-28-07392-s001.zip › molecules-2602370-supplementary.pdf]

Supplementary Information

Inhibitory Activity of 4-Benzylidene Oxazolones Derivatives of Cinnamic Acid on Human Acetylcholinesterase and Cognitive Improvements in a Mouse Model

Alma Marisol Ramírez-Ruiz [1,2], Martha Elena Ávila-Cossío [3], Arturo Estolano-Cobián [2], José Manuel Cornejo-Bravo [2], Ana Laura Martinez [1], Iván Córdova-Guerrero [2], Bibiana Roselly Cota-Ramírez [2], Krysta Paola Carranza-Ambriz [1], Ignacio A. Rivero [3,*] and Aracely Serrano-Medina [1,2,*]

1 Facultad de Medicina y Psicología, Universidad Autónoma de Baja California, Calzada Universidad 14418, Parque Industrial Internacional, Tijuana 22424, BC, Mexico; ana.laura.martinez.martinez@uabc.edu.mx (A.L.M.); krysta.carranza@uabc.edu.mx (K.P.C.-A.)

2 Facultad de Ciencias Químicas e Ingeniería, Universidad Autónoma de Baja California, Calzada Universidad 14418, Parque Industrial Internacional, Tijuana 22424, BC, Mexico; arturo.estolano@uabc.edu.mx (A.E.-C.); jmcornejo@uabc.edu.mx (J.M.C.-B.); icordova@uabc.edu.mx (I.C.-G.); bibiana.cota@uabc.edu.mx (B.R.C.-R.)

3 Centro de Graduados e Investigación en Química, Tecnológico Nacional de Mexico/Instituto Tecnológico de Tijuana, Tijuana 22510, BC, Mexico; myf91@hotmail.com

* Correspondence: irivero@tectijuana.mx (I.A.R.); serrano.aracely@uabc.edu.mx (A.S.-M.)

**4-((*Z*)-benzylidene)-2-(*E*)-styryl)oxazol-5(4H)-one**	S3
Figure S1. ^{1}H (400 MHz) NMR spectrum	S3
Figure S2. ^{13}C (100 MHz) NMR spectrum	S4
Figure S3. EI-MS spectrum	S5
Figure S4. HRMS spectrum	S6
Figure S5. FT-IR (ATR) spectrum	S7
**4-((*Z*)-4-methylbenzylidene)-2-((*E*)-styryl)oxazol-5(4H)-one**	S8
Figure S6. ^{1}H (400 MHz) NMR spectrum	S8
Figure S7. ^{13}C (100 MHz) NMR spectrum	S9
Figure S8. EI-MS spectrum	S10
Figure S9. HRMS spectrum	S11
Figure S10. FT-IR (ATR) spectrum	S12
**4-((*Z*)-4-chlorobenzylidene)-2-(*E*)-styryl)oxazol-5(4H)-one**	S13
Figure S11. ^{1}H (400 MHz) NMR spectrum	S13
Figure S12. ^{13}C (100 MHz) NMR spectrum	S14
Figure S13. EI-MS spectrum	S15
Figure S14. HRMS spectrum	S16
Figure S15. FT-IR (ATR) spectrum	S17
**4-((*Z*)-4-methoxybenzylidene)-2-(*E*)-styryl)oxazol-5(4H)-one**	S18
Figure S16. ^{1}H (400 MHz) NMR spectrum	S18
Figure S17. ^{13}C (100 MHz) NMR spectrum	S19
Figure S18. EI-MS spectrum	S20
Figure S19. HRMS spectrum	S21

Figure S20. FT-IR (ATR) spectrum	S22
**3-((*Z*)-(5-oxo-2-((*E*)-styryl)oxazol-4(5H)-ylidene)methyl)phenyl acetate**	S23
Figure S21. ^{1}H (400 MHz) NMR spectrum	S23
Figure S22. ^{13}C (100 MHz) NMR spectrum	S24
Figure S23. EI-MS spectrum	S25
Figure S24. HRMS spectrum	S26
Figure S25. FT-IR (ATR) spectrum	S27
**4-((*Z*)-(5-oxo-2((*E*)-styryl)oxazol-4(5H)-ylidine)methyl)phenyl acetate**	S28
Figure S26. ^{1}H (400 MHz) NMR spectrum	S28
Figure S27. ^{13}C (100 MHz) NMR spectrum	S29
Figure S28. EI-MS spectrum	S30
Figure S29. HRMS spectrum	S31
Figure S30. FT-IR (ATR) spectrum	S32
**2-hydroxy-3-((*Z*)-(5-oxo-2-((*E*)-styryl)oxazol-4(5H)-ylidene)methyl)phenyl acetate**	S33
Figure S31. ^{1}H (400 MHz) NMR spectrum	S33
Figure S32. ^{13}C (100 MHz) NMR spectrum	S34
Figure S33. EI-MS spectrum	S35
Figure S34. HRMS spectrum	S36
Figure S35. FT-IR (ATR) spectrum	S37

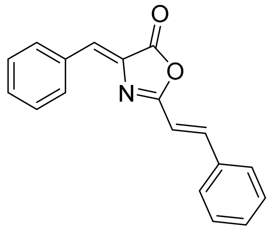

4-((*Z*)-benzylidene)-2-(*E*)-styryl)oxazol-5(4H)-one (1)

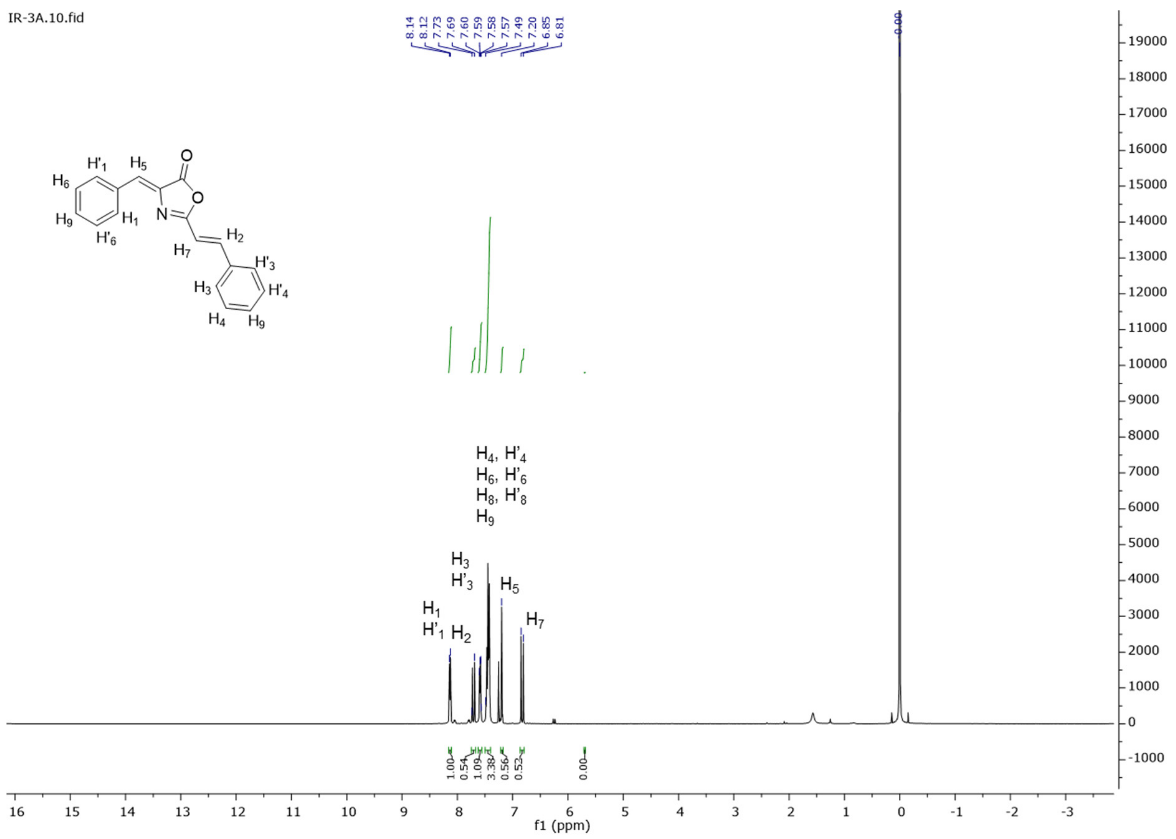

Figure S1. ^{1}H (400 MHz) NMR spectrum for compound **1** in $CDCl_3$ at 298 K.

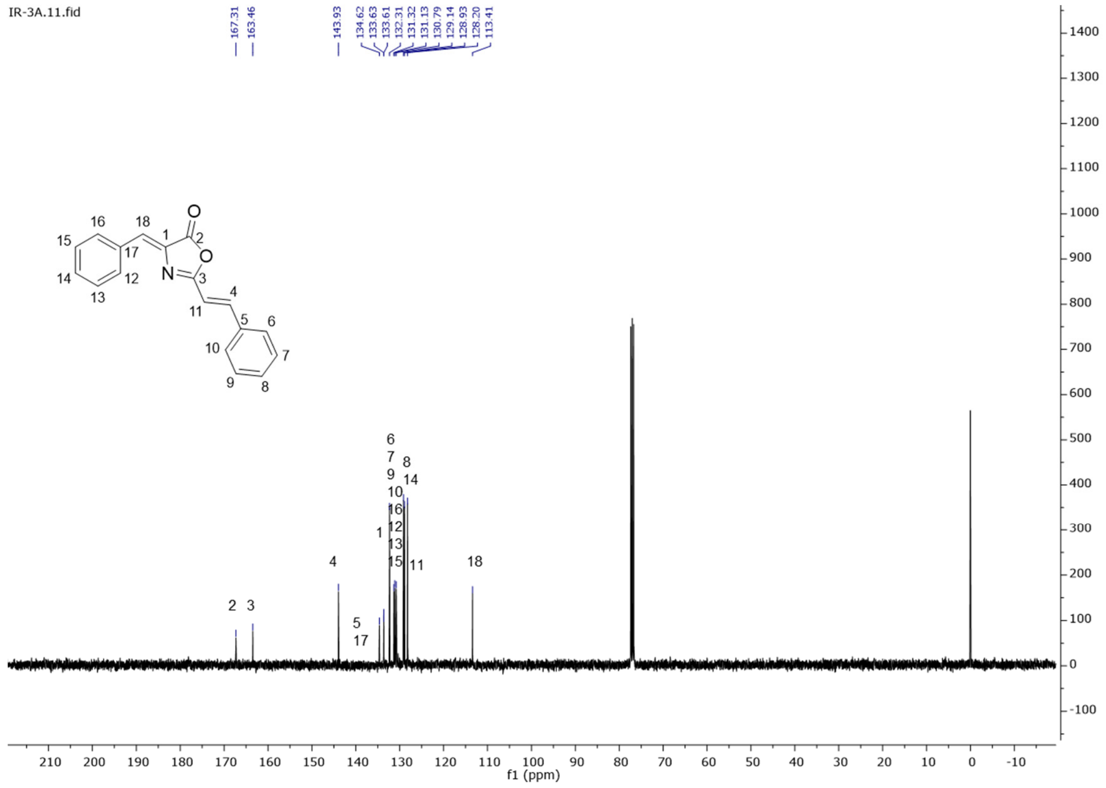

Figure S2. ^{13}C (100 MHz) NMR spectrum for compound **1** at 298 K.

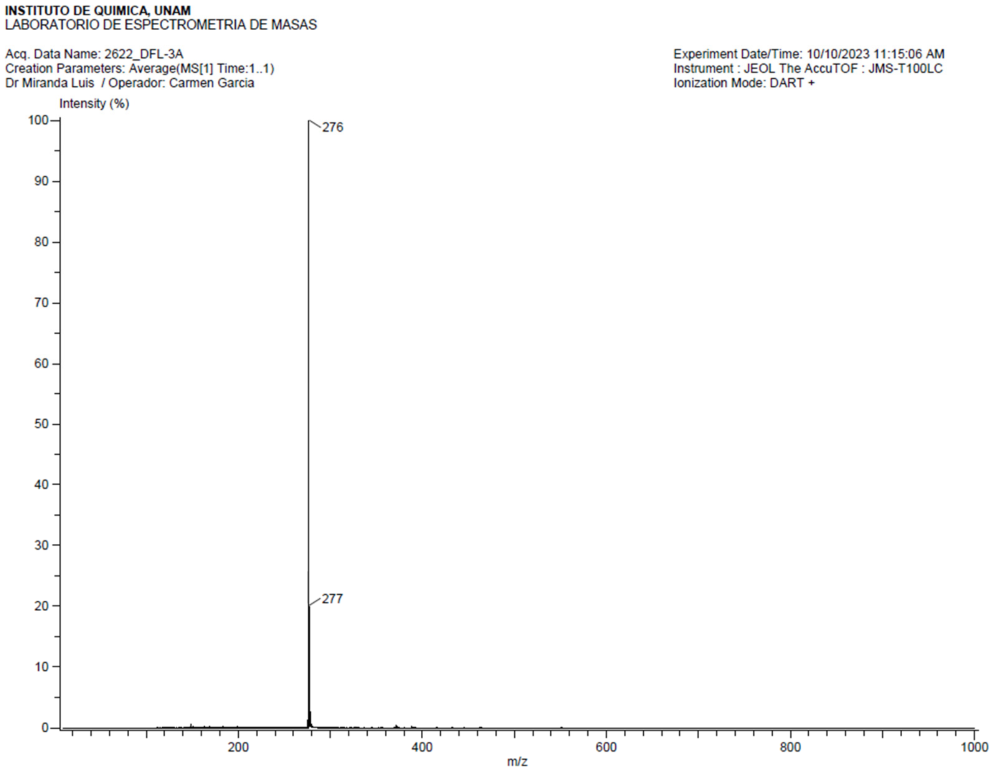

Figure S3. EI-MS spectrum for compound **1**.

Data:2622_DFL-3A
Sample Name:Dr Miranda Luis / Operador: Carmen Garcia
Description:
Ionization Mode:ESI+
History:Determine m/z[Peak Detect[Centroid,30,Area];Correct Base[];Smooth[5]];Correct Base[5.0%];Average(MS[...

Acquired:10/10/2023 11:15:06 AM
Operator:AccuTOF
Mass Calibration data:cal-PEG-600-CG
Created:10/17/2023 6:05:42 PM
Created by:AccuTOF

Charge number:1
Tolerance:3.00(mmu)
Unsaturation Number:0.0 .. 50.0 (Fraction:.5)
Element:^{12}C:0 .. 30, ^{1}H:0 .. 60, ^{14}N:0 .. 3, ^{16}O:0 .. 4

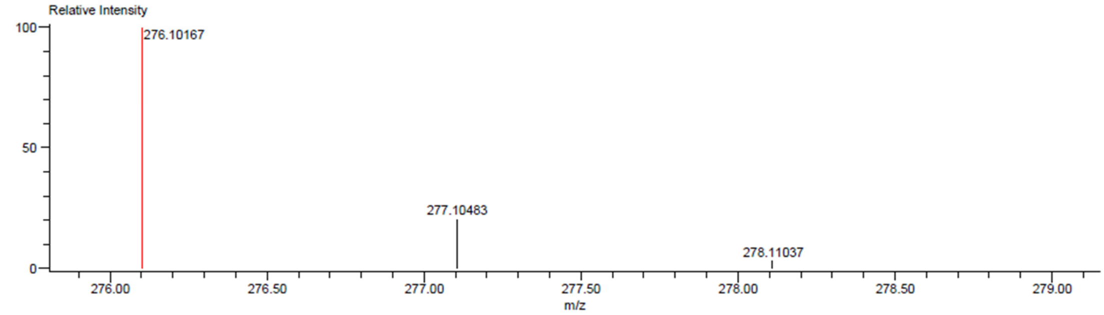

Mass	Intensity	Calc. Mass	Mass Difference (mmu)	Mass Difference (ppm)	Possible Formula	Unsaturation Number
276.10167	1327681.42	276.10245	-0.78	-2.84	$^{12}C_{18}{}^{1}H_{14}{}^{14}N_{1}{}^{16}O_{2}$	12.5

Figure S4. HRMS spectrum for compound **1**.

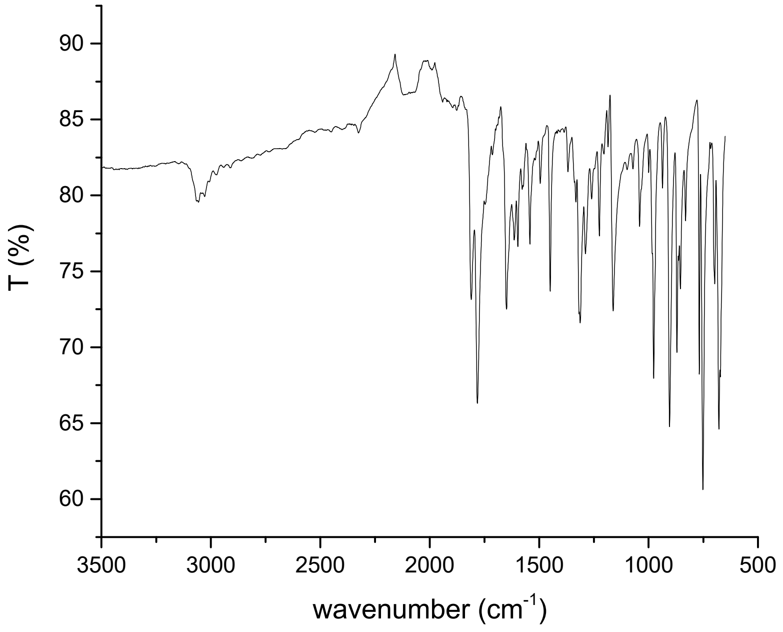

Figure S5. FT-IR (ATR) spectrum for compound **1**.

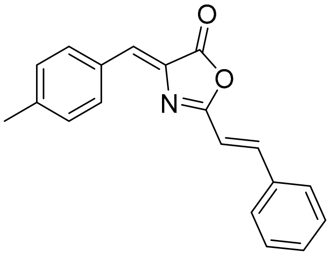

4-((*Z*)-4-methylbenzylidene)-2-((*E*)-styryl)oxazol-5(4H)-one (2)

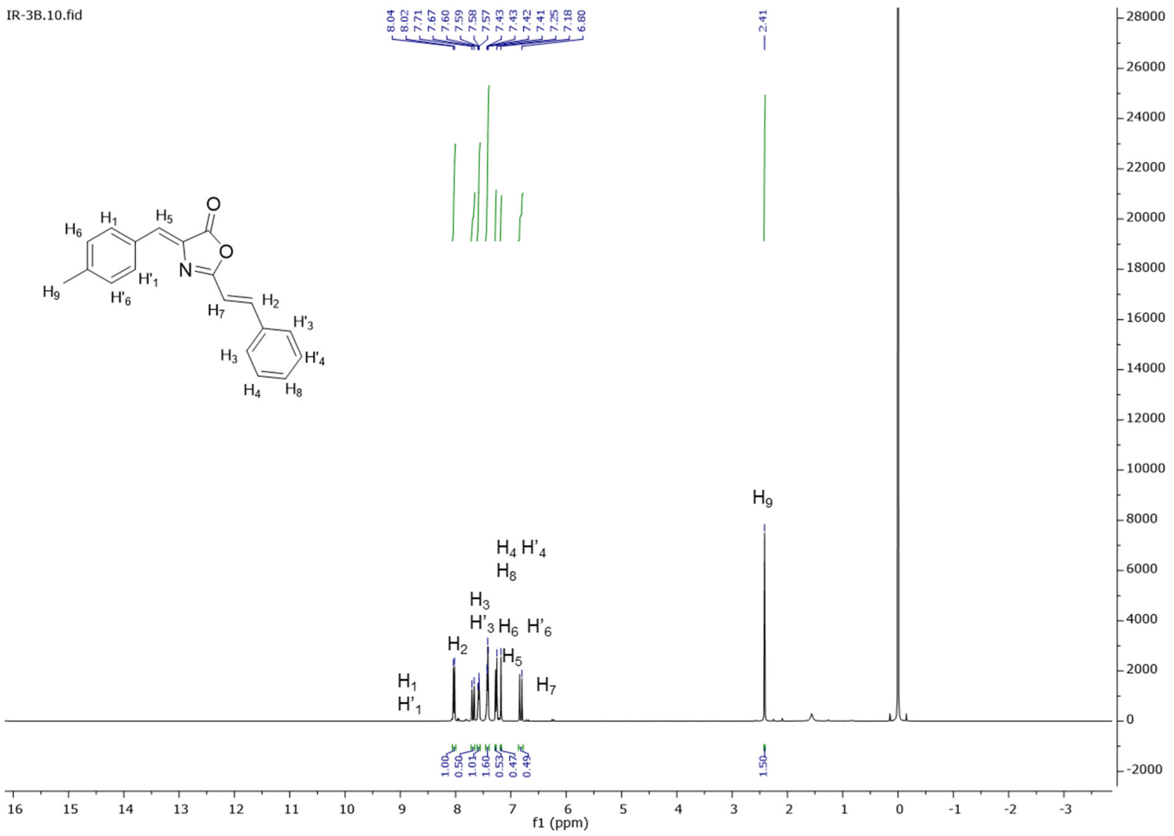

Figure S6. ^{1}H (400 MHz) NMR spectrum for compound **2** in $CDCl_3$ at 298 K.

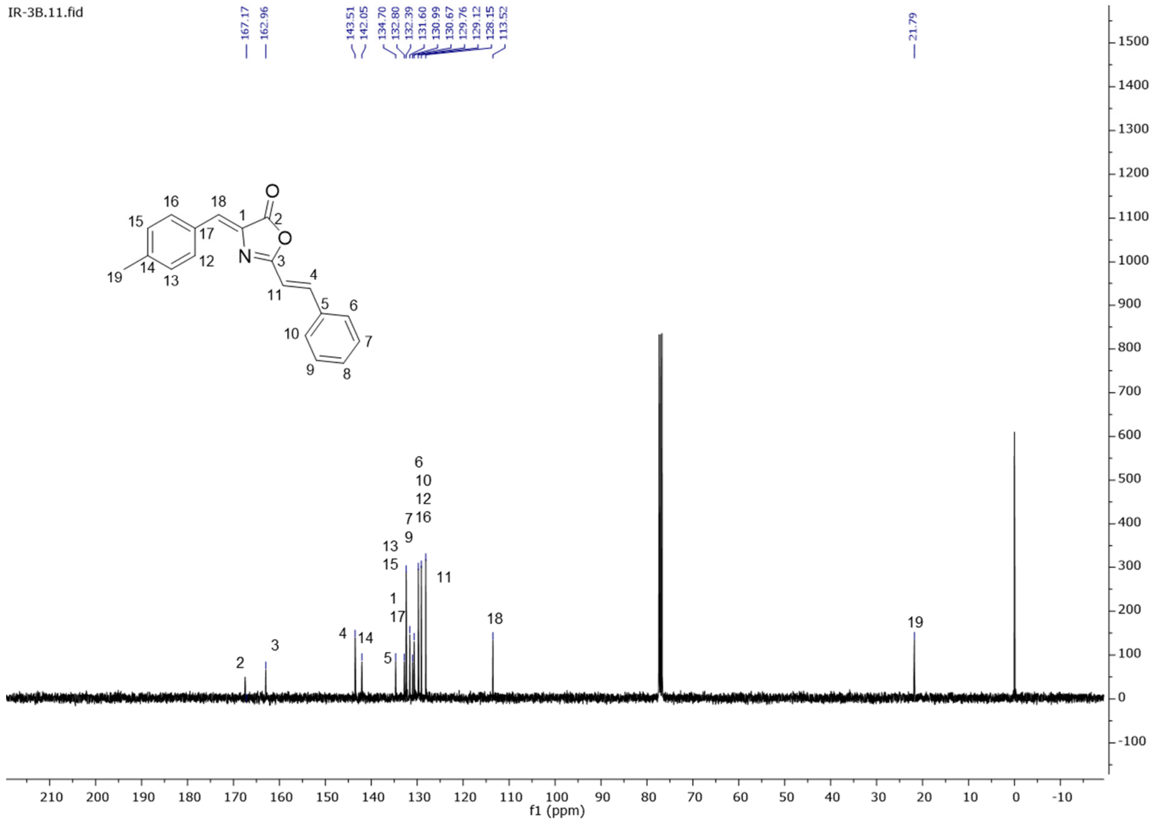

Figure S7. ^{13}C (100 MHz) NMR spectrum for compound **2** at 298

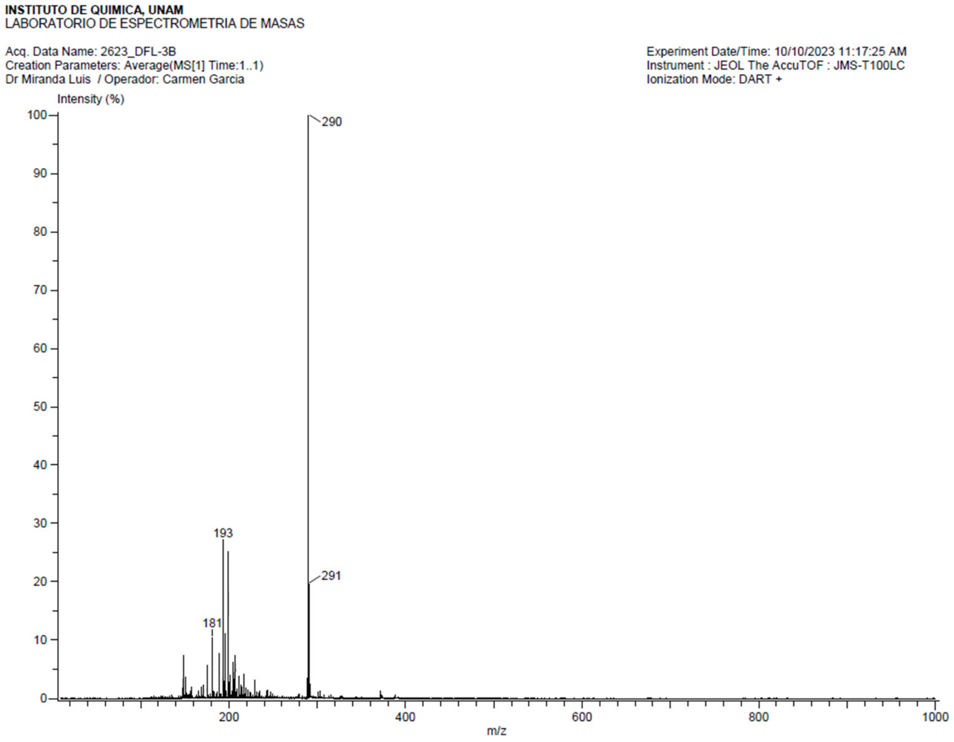

Figure S8. EI-MS spectrum for compound **2**.

Data:2623_DFL-3B
Sample Name:Dr Miranda Luis / Operador: Carmen Garcia
Description:
Ionization Mode:ESI+
History:Determine m/z[Peak Detect[Centroid,30,Area];Correct Base[];Smooth[5]];Correct Base[5.0%];Average(MS[...

Acquired:10/10/2023 11:17:25 AM
Operator:AccuTOF
Mass Calibration data:cal-PEG-600-CG
Created:10/17/2023 6:17:42 PM
Created by:AccuTOF

Charge number:1
Tolerance:3.00(mmu)
Unsaturation Number:0.0 .. 50.0 (Fraction:.5)
Element:^{12}C:0 .. 30, ^{1}H:0 .. 60, ^{14}N:0 .. 3, ^{16}O:0 .. 3

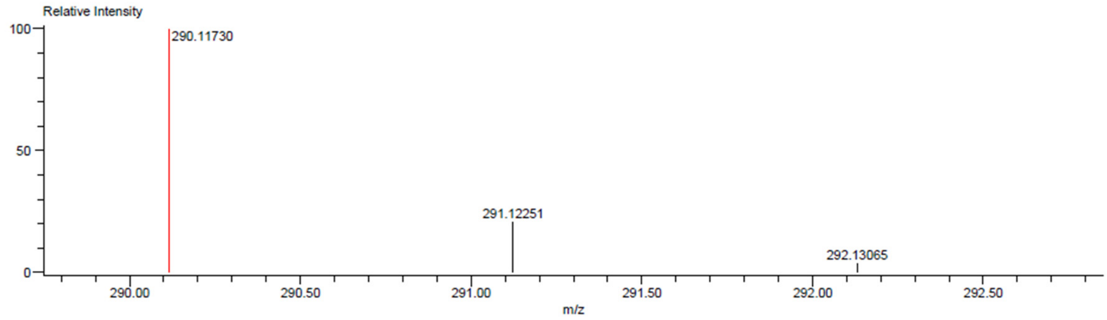

Mass	Intensity	Calc. Mass	Mass Difference (mmu)	Mass Difference (ppm)	Possible Formula	Unsaturation Number
290.11730	265755.79	290.11810	-0.81	-2.78	$^{12}C_{19}{}^{1}H_{16}{}^{14}N_{1}{}^{16}O_{2}$	12.5

Figure S9. HRMS spectrum for compound **2**.

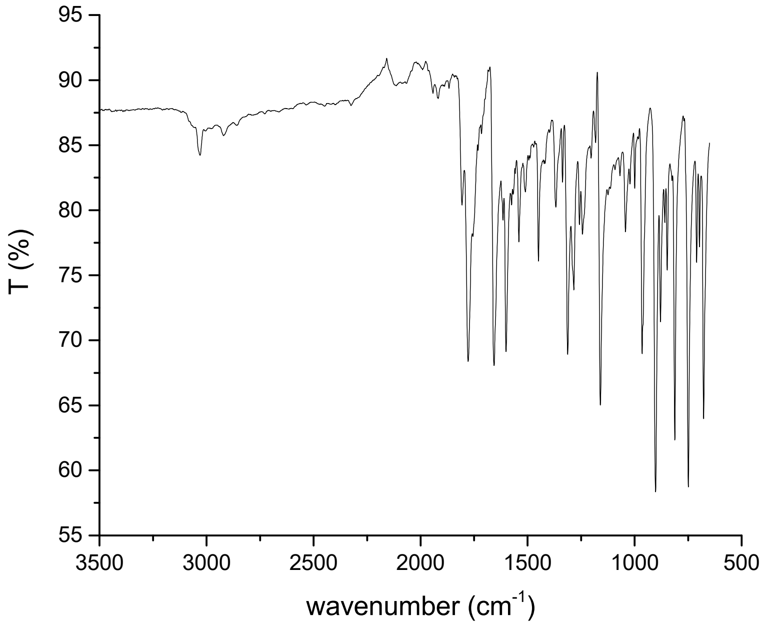

Figure S10. FT-IR (ATR) spectrum for compound **2**.

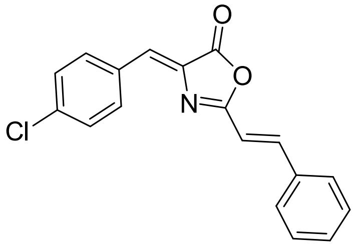

4-((*Z*)-4-chlorobenzylidene)-2-(*E*)-styryl)oxazol-5(4H)-one (3)

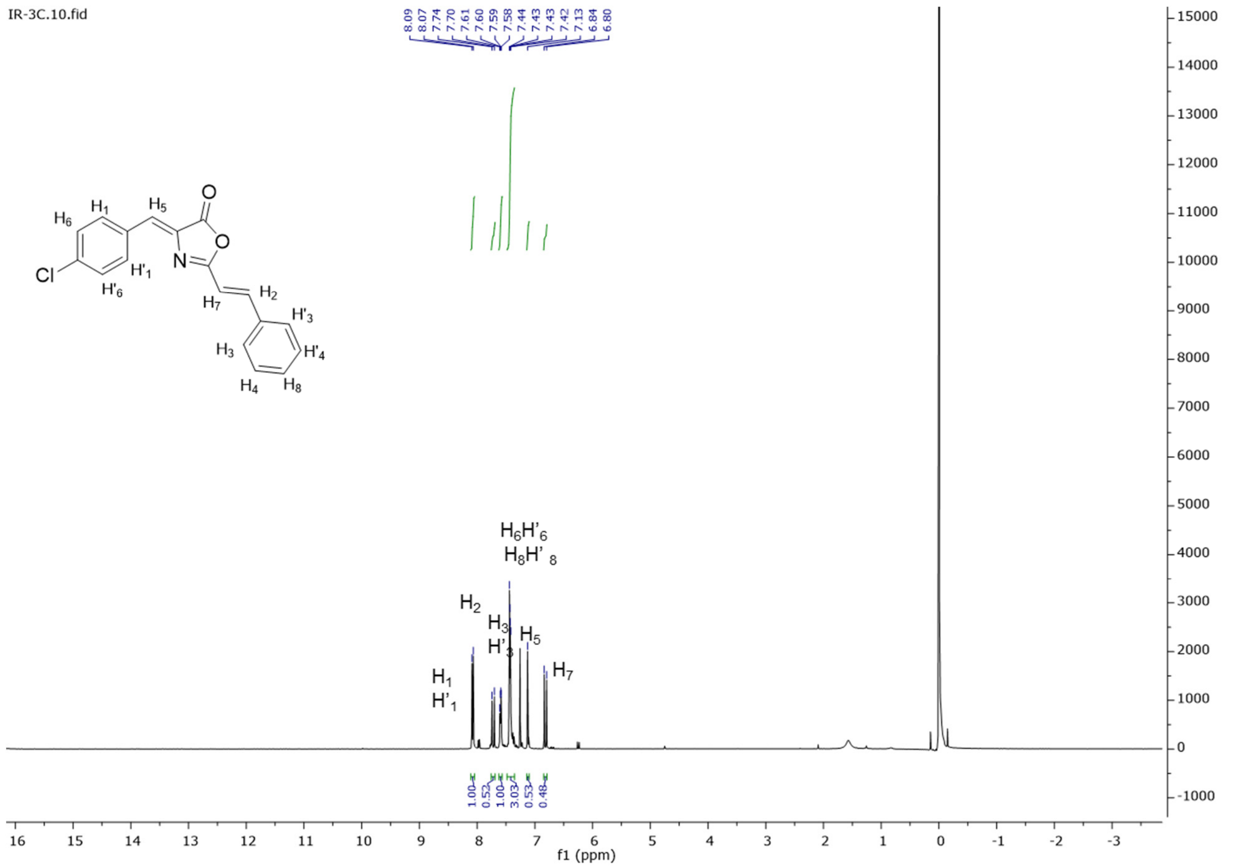

Figure S11. ^{1}H (400 MHz) NMR spectrum for compound **3** in $CDCl_3$ at 298 K.

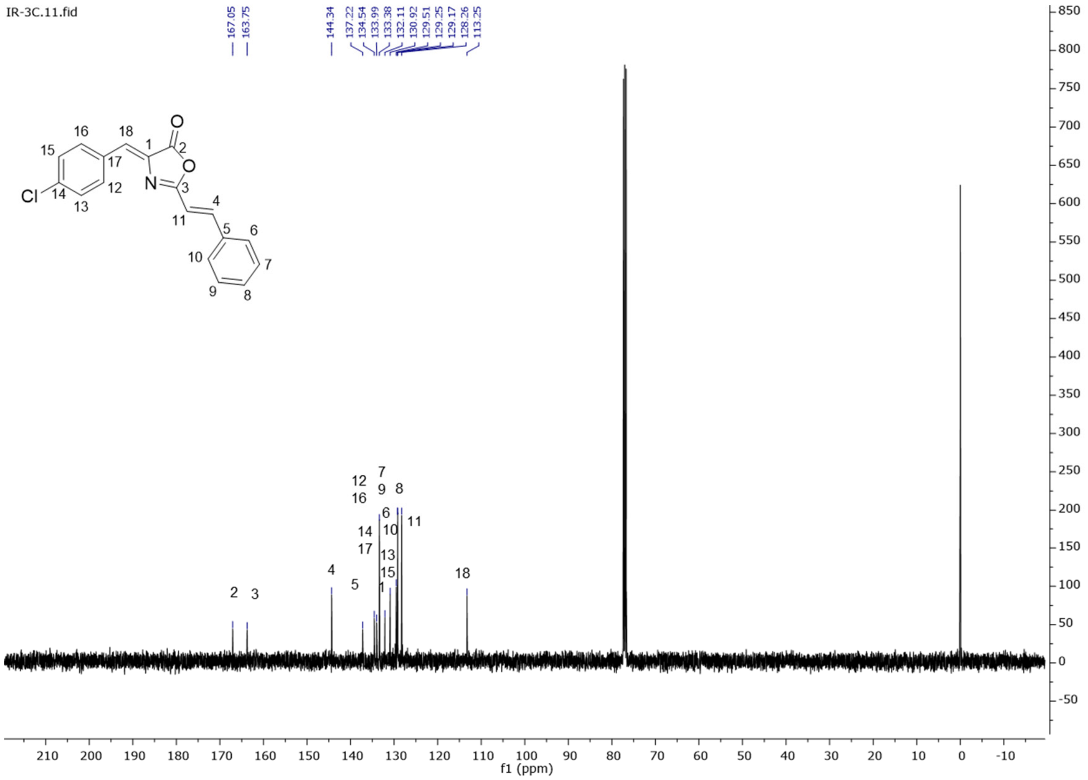

Figure S12. ^{13}C (100 MHz) NMR spectrum for compound **3** at 298 K.

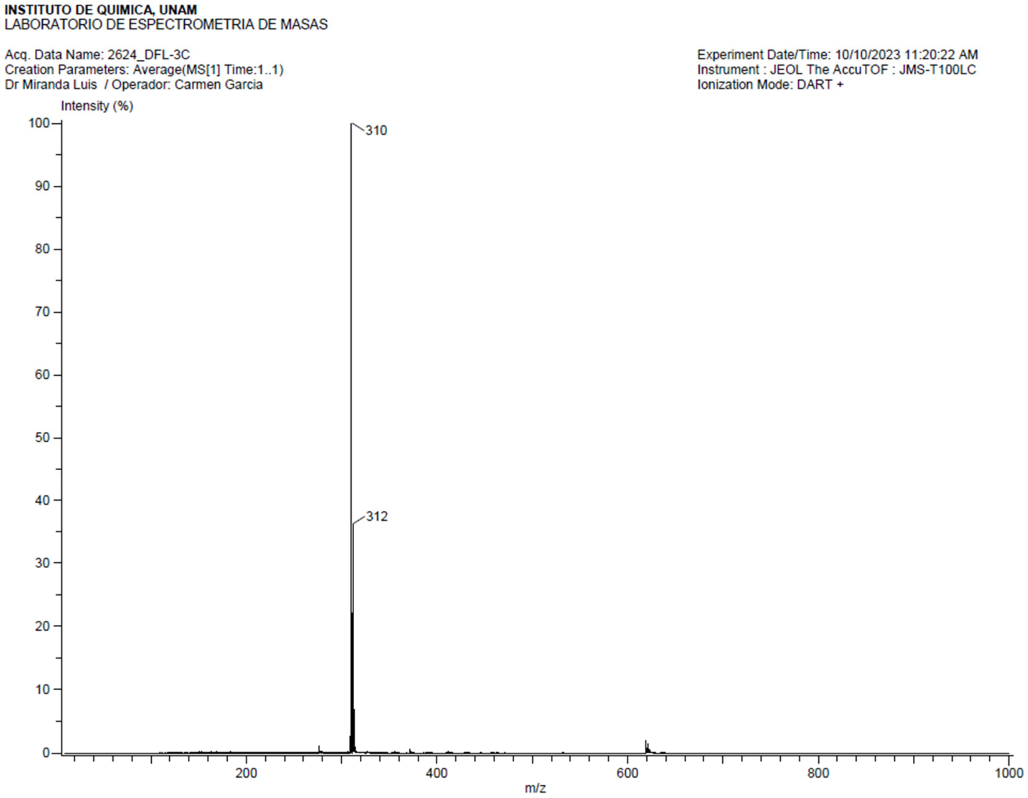

Figure S13. EI-MS spectrum for compound **3**.

Data:2624_DFL-3C
Sample Name:Dr Miranda Luis / Operador: Carmen Garcia
Description:
Ionization Mode:ESI+
History:Determine m/z[Peak Detect[Centroid,30,Area];Correct Base[];Smooth[5]];Correct Base[5.0%];Average(MS[...

Acquired:10/10/2023 11:20:22 AM
Operator:AccuTOF
Mass Calibration data:cal-PEG-600-CG
Created:10/17/2023 6:26:09 PM
Created by:AccuTOF

Charge number:1
Tolerance:3.00(mmu)
Unsaturation Number:0.0 .. 50.0 (Fraction:.5)
Element:^{12}C:0 .. 30, ^{1}H:0 .. 60, ^{35}Cl:1 .. 1, ^{14}N:0 .. 3, ^{16}O:0 .. 3

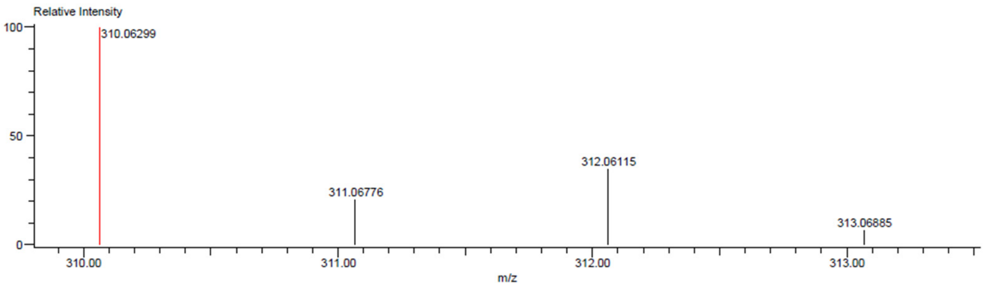

Mass	Intensity	Calc. Mass	Mass Difference (mmu)	Mass Difference (ppm)	Possible Formula	Unsaturation Number
310.06299	646478.23	310.06348	-0.50	-1.60	$^{12}C_{18}{}^{1}H_{13}{}^{35}Cl_{1}{}^{14}N_{1}{}^{16}O_{2}$	12.5

Figure S14. HRMS spectrum for compound **3**.

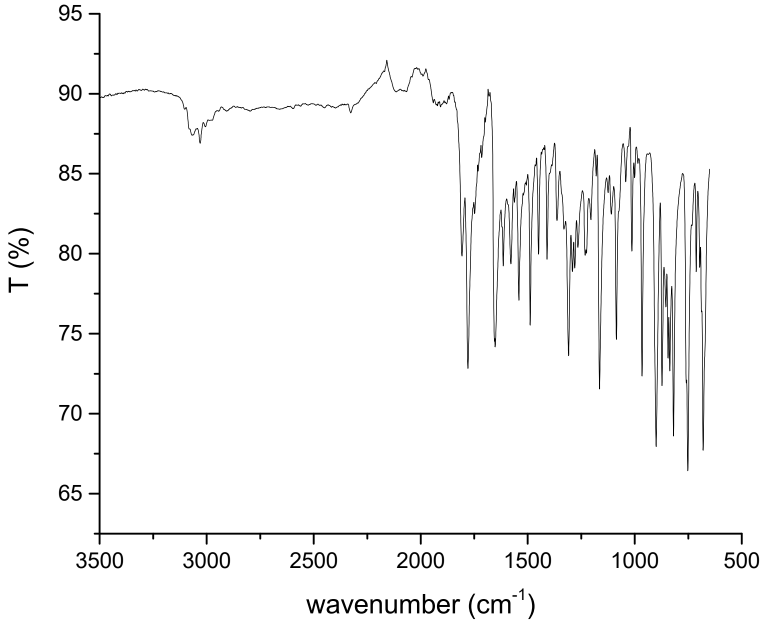

Figure S15. FT-IR (ATR) spectrum for compound **3**.

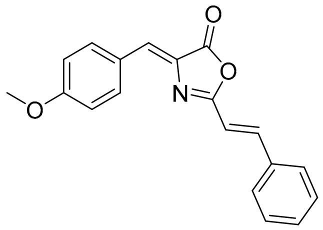

4-((*Z*)-4-methoxybenzylidene)-2-(*E*)-styryl)oxazol-5(4H)-one (4)

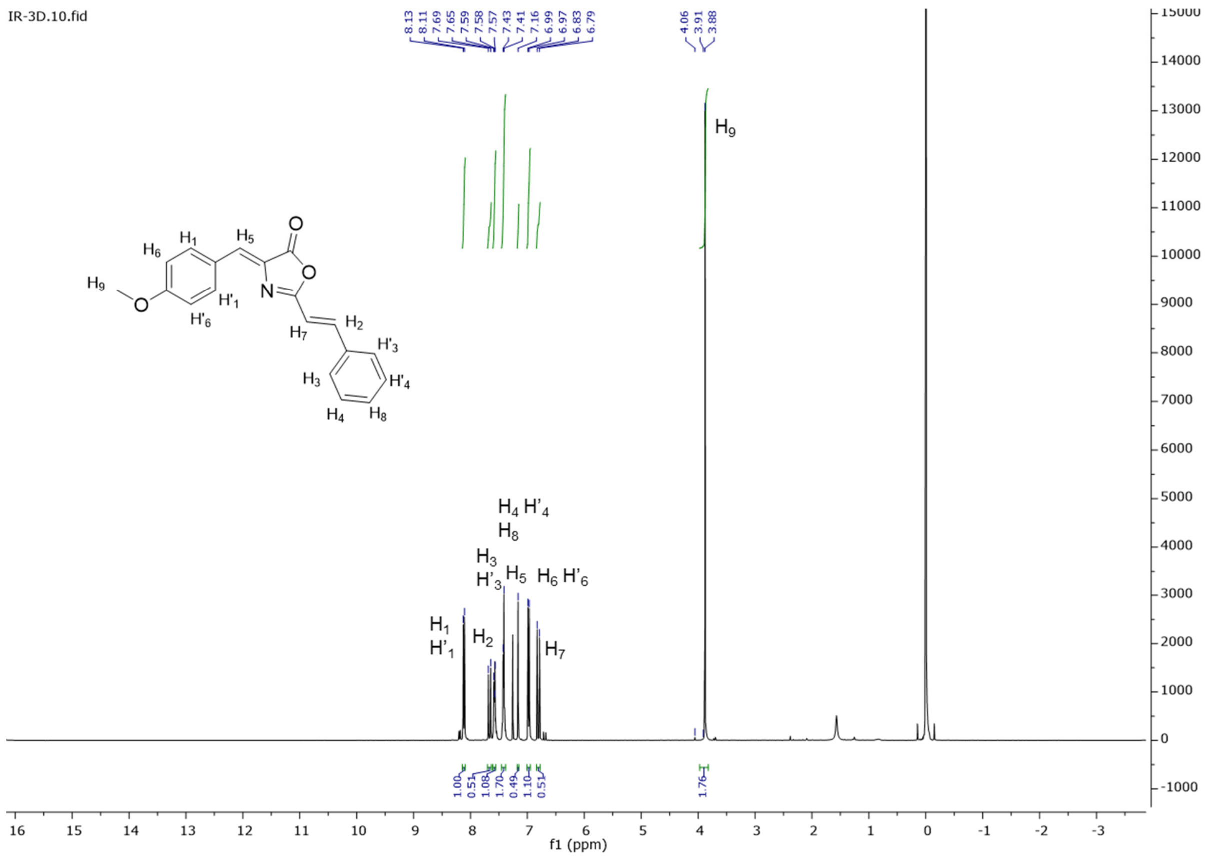

Figure S16. ^{1}H (400 MHz) NMR spectrum for compound **4** in $CDCl_3$ at 298 K.

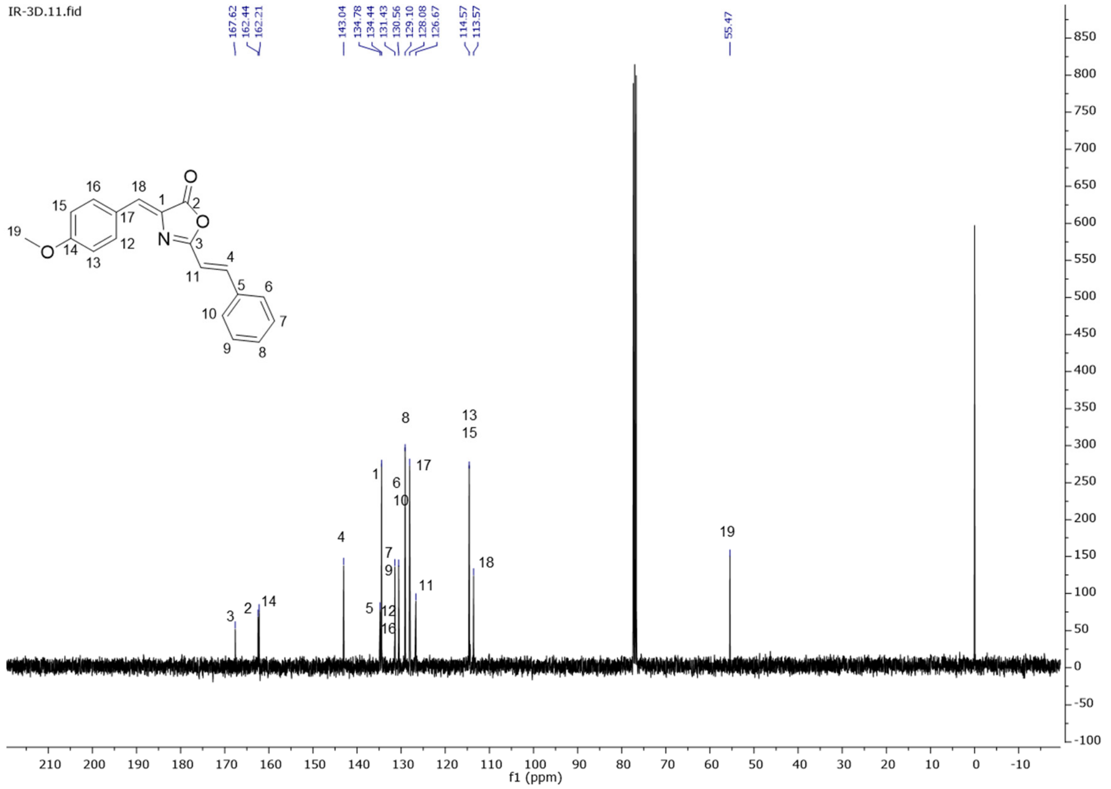

Figure S17. ^{13}C (100 MHz) NMR spectrum for compound **4** at 298 K.

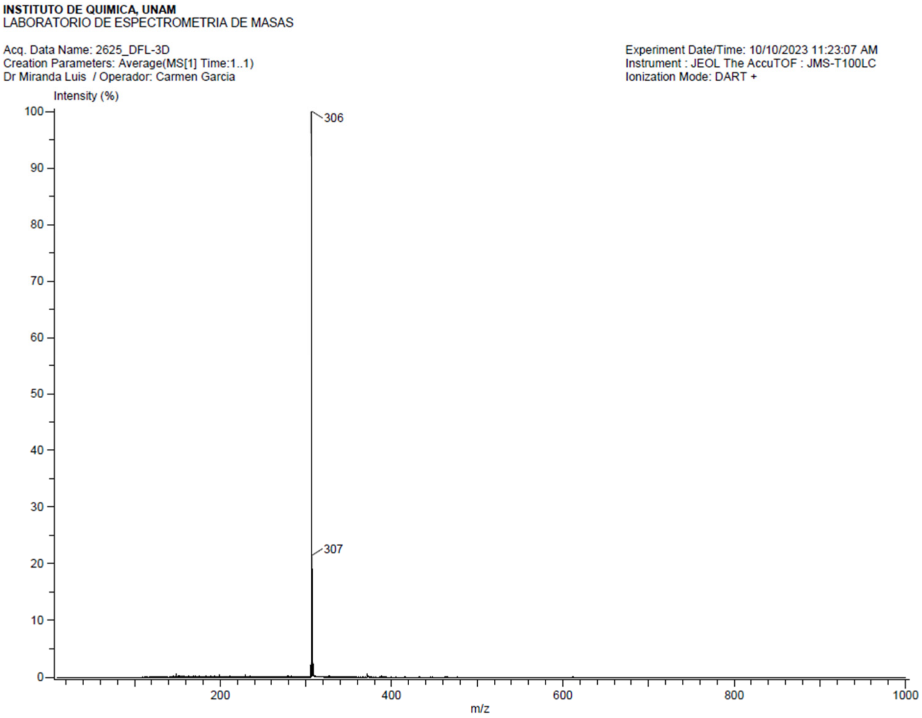

Figure S18. EI-MS spectrum for compound **4**.

Data:2625_DFL-3D
Sample Name:Dr Miranda Luis / Operador: Carmen Garcia
Description:
Ionization Mode:ESI+
History:Determine m/z[Peak Detect[Centroid,30,Area];Correct Base[];Smooth[5]];Correct Base[5.0%];Average(MS[...

Acquired:10/10/2023 11:23:07 AM
Operator:AccuTOF
Mass Calibration data:cal-PEG-600-CG
Created:10/17/2023 6:30:36 PM
Created by:AccuTOF

Charge number:1
Element:^{12}C:0 .. 30, ^{1}H:0 .. 60, ^{14}N:0 .. 3, ^{16}O:0 .. 4

Tolerance:3.00(mmu)

Unsaturation Number:0.0 .. 50.0 (Fraction:.5)

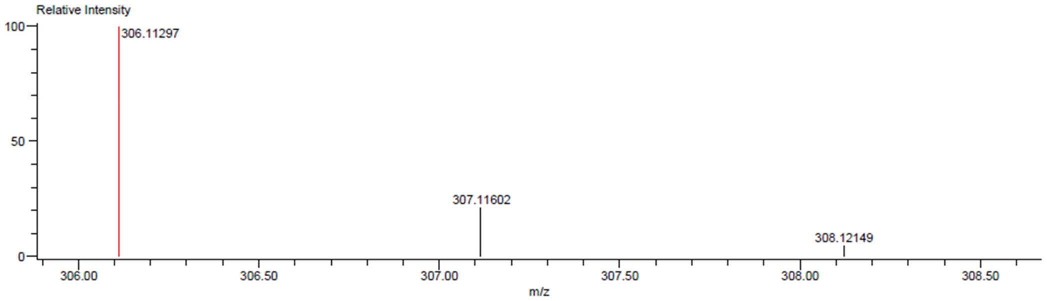

Mass	Intensity	Calc. Mass	Mass Difference (mmu)	Mass Difference (ppm)	Possible Formula	Unsaturation Number
306.11297	999587.55	306.11302	-0.05	-0.17	$^{12}C_{19}{}^{1}H_{16}{}^{14}N_{1}{}^{16}O_{3}$	12.5

Figure S19. HRMS spectrum for compound **4**.

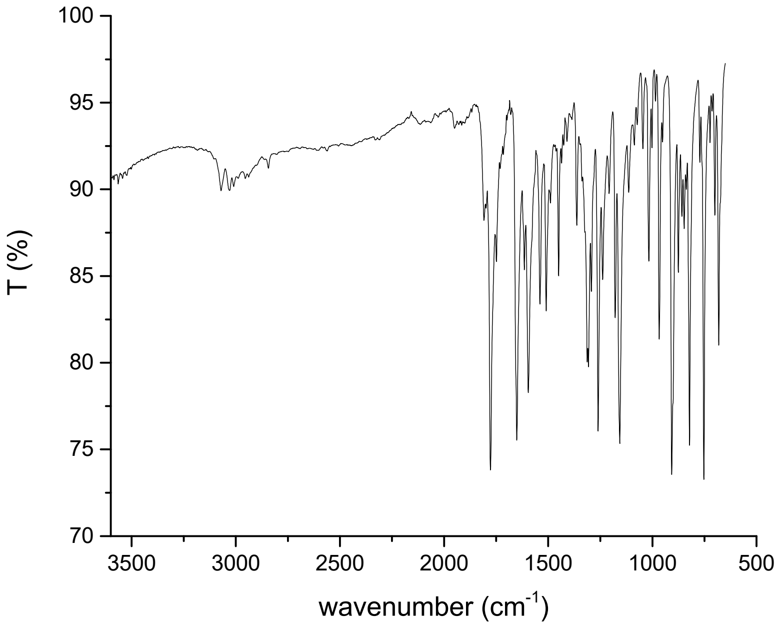

Figure S20. FT-IR (ATR) spectrum for compound **4**.

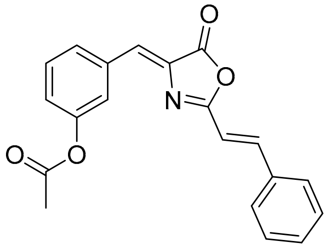

3-((*Z*)-(5-oxo-2-((*E*)-styryl)oxazol-4(5H)-ylidene)methyl)phenyl acetate (5)

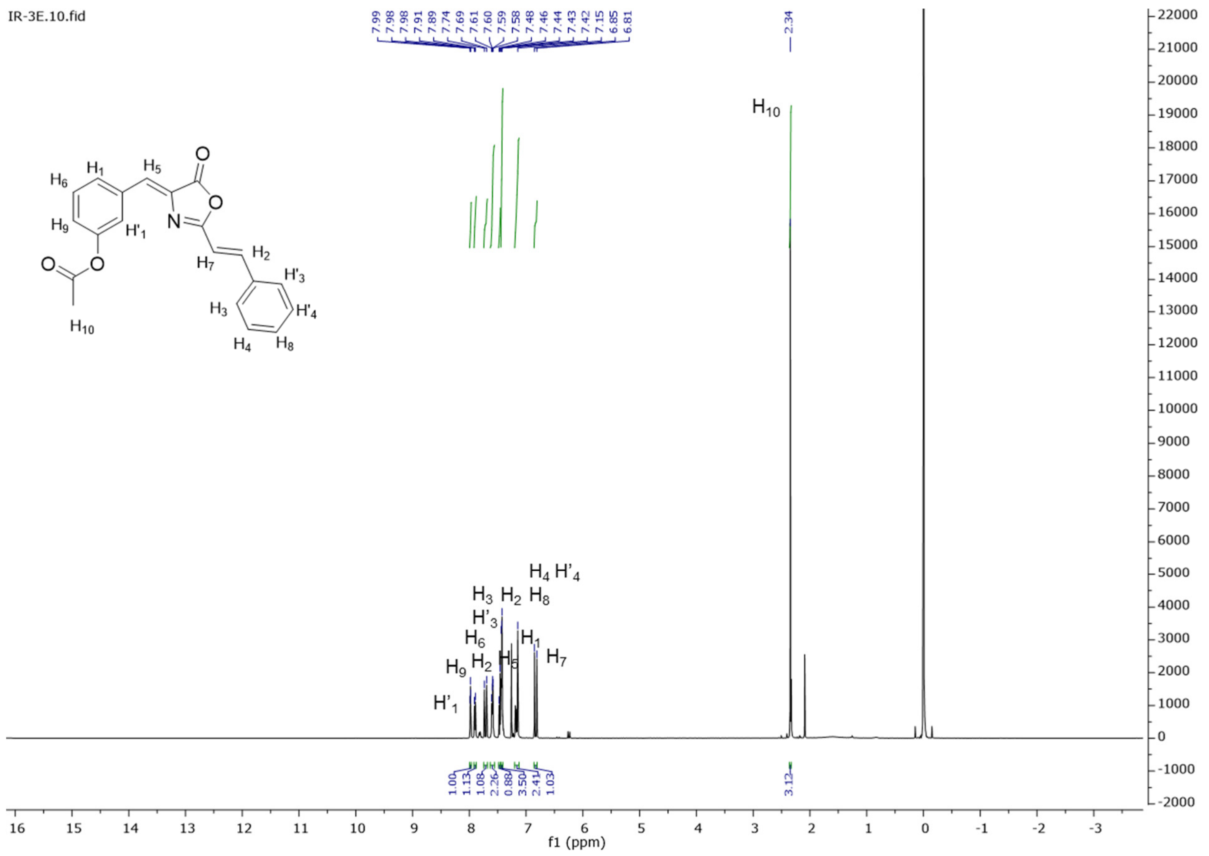

Figure S21. ^{1}H (400 MHz) NMR spectrum for compound **5** in $CDCl_3$ at 298 K.

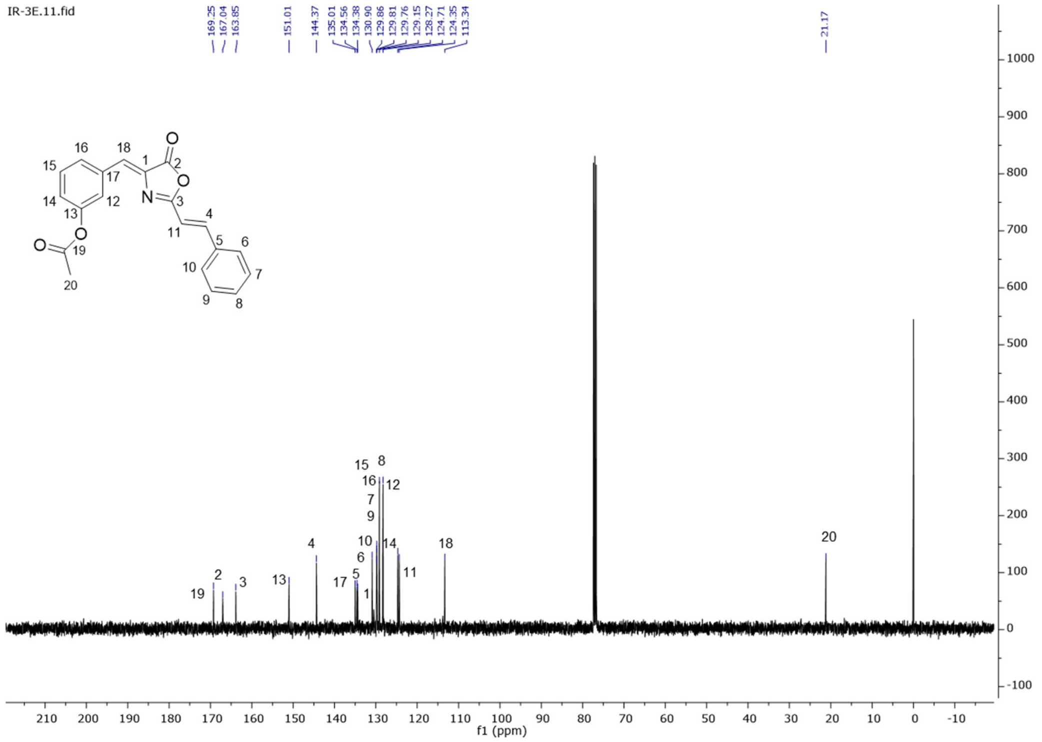

Figure S22. ^{13}C (100 MHz) NMR spectrum for compound **5** at 298 K.

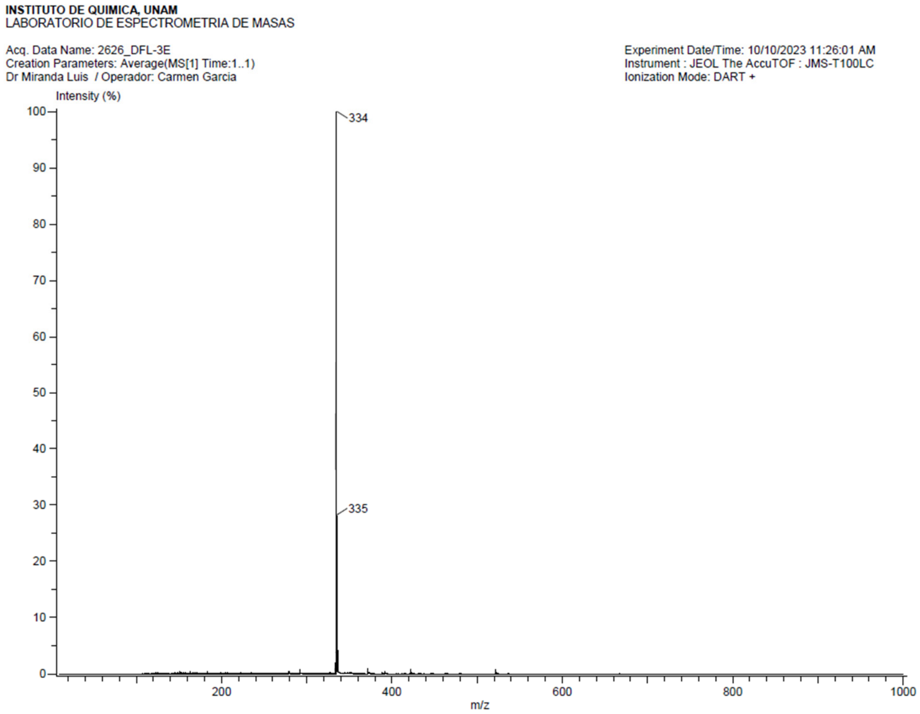

Figure S23. EI-MS spectrum for compound **5**.

Data:2626_DFL-3E
Sample Name:Dr Miranda Luis / Operador: Carmen Garcia
Description:
Ionization Mode:ESI+
History:Determine m/z[Peak Detect[Centroid,30,Area];Correct Base[];Smooth[5]];Correct Base[5.0%];Average(MS[...

Acquired:10/10/2023 11:26:01 AM
Operator:AccuTOF
Mass Calibration data:cal-PEG-600-CG
Created:10/17/2023 6:34:34 PM
Created by:AccuTOF

Charge number:1
Tolerance:3.00(mmu)
Unsaturation Number:0.0 .. 50.0 (Fraction:.5)
Element:^{12}C:0 .. 30, ^{1}H:0 .. 60, ^{14}N:0 .. 3, ^{16}O:0 .. 4

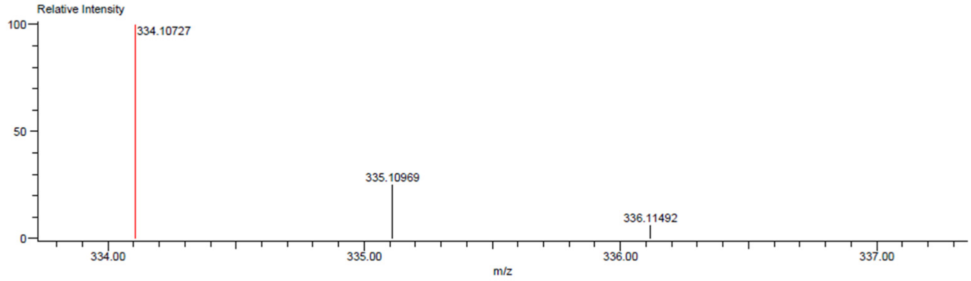

Mass	Intensity	Calc. Mass	Mass Difference (mmu)	Mass Difference (ppm)	Possible Formula	Unsaturation Number
334.10727	3207963.78	334.10793	-0.67	-2.00	$^{12}C_{20}{}^{1}H_{16}{}^{14}N_{1}{}^{16}O_{4}$	13.5

Figure S24. HRMS spectrum for compound **5**.

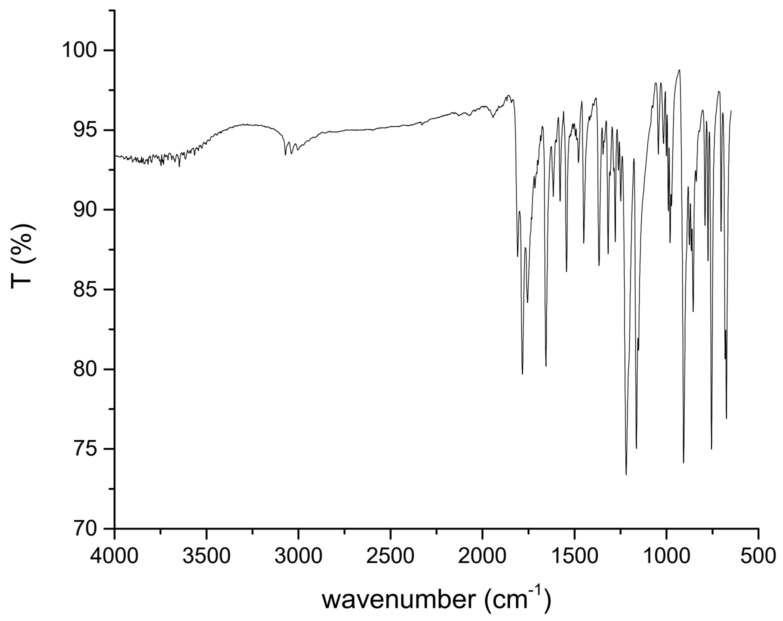

Figure S25. FT-IR (ATR) spectrum for compound **5**.

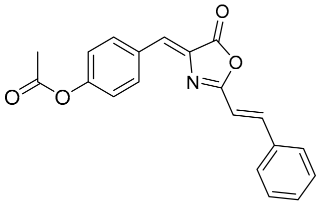

4-((*Z*)-(5-oxo-2((*E*)-styril)oxazol-4(5H)-ylidine)methyl)phenyl acetate (6)

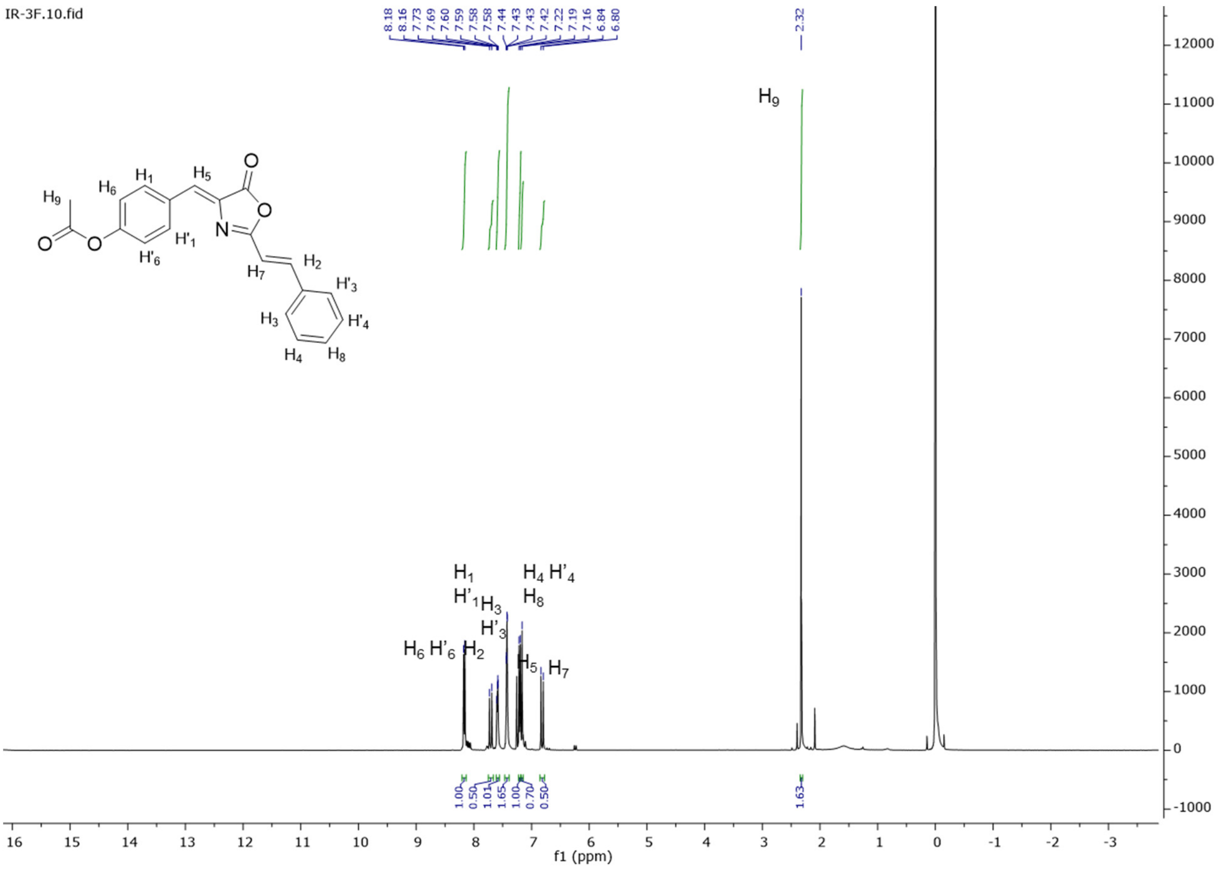

Figure S26. ^{1}H (400 MHz) NMR spectrum for compound **6** in $CDCl_3$ at 298 K.

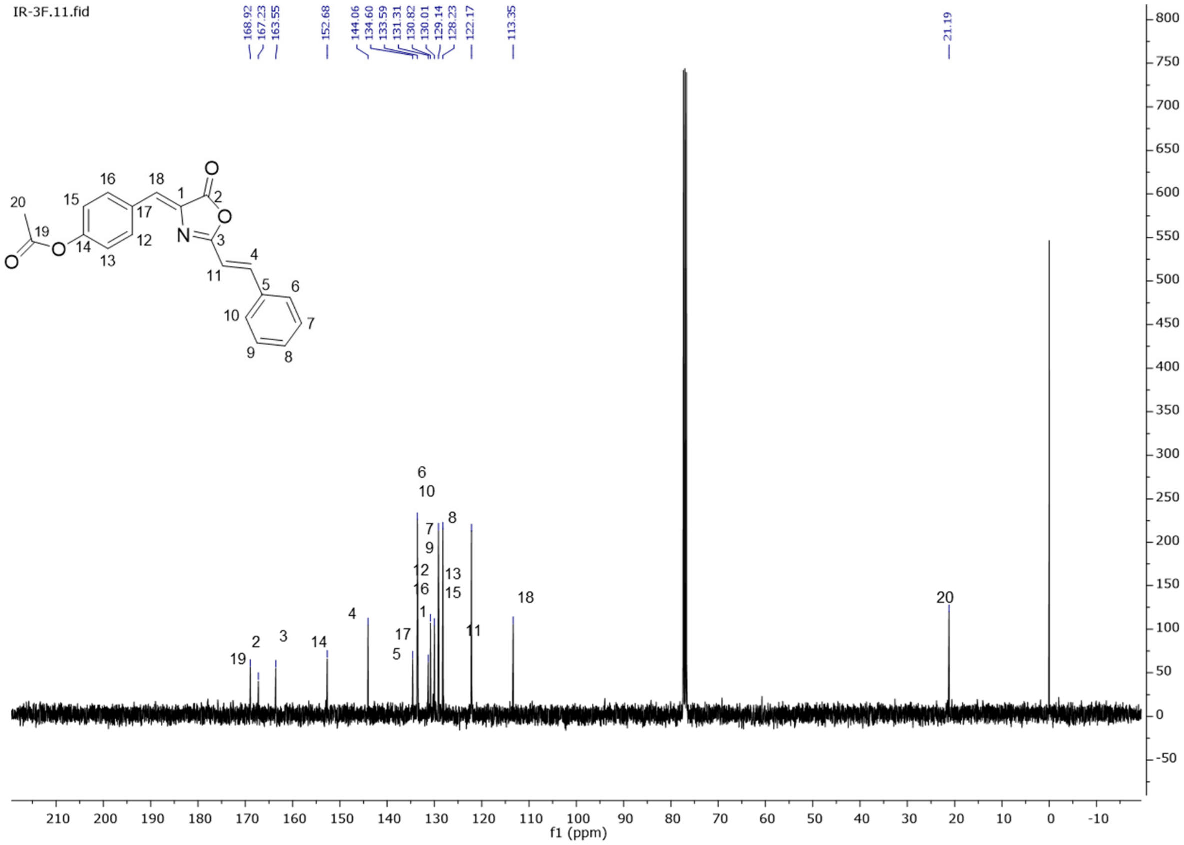

Figure S27. ^{13}C (100 MHz) NMR spectrum for compound **6** in $CDCl_3$ at 298 K.

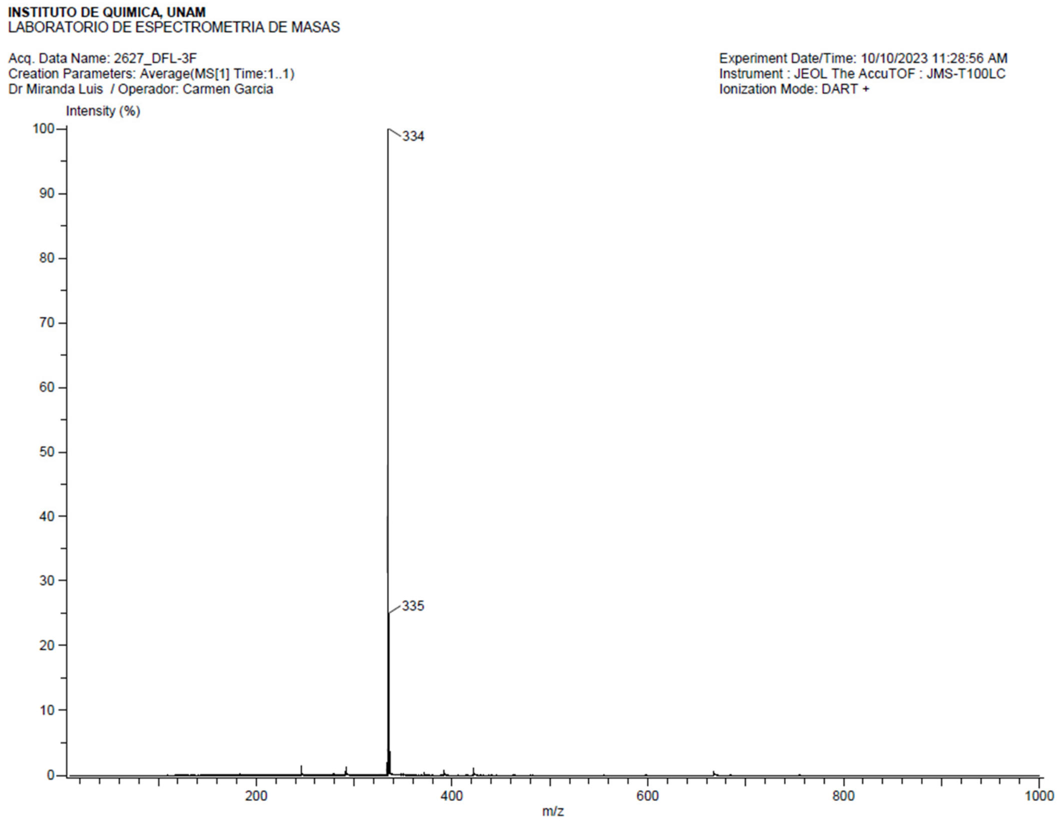

Figure S28. EI-MS spectrum for compound **6**.

Data:2627_DFL-3F
Sample Name:Dr Miranda Luis / Operador: Carmen Garcia
Description:
Ionization Mode:ESI+
History:Determine m/z[Peak Detect[Centroid,30,Area];Correct Base[];Smooth[5]];Correct Base[5.0%];Average(MS[...

Acquired:10/10/2023 11:28:56 AM
Operator:AccuTOF
Mass Calibration data:cal-PEG-600-CG
Created:10/17/2023 6:39:22 PM
Created by:AccuTOF

Charge number:1
Tolerance:3.00(mmu)
Unsaturation Number:0.0 .. 50.0 (Fraction:.5)
Element:^{12}C:0 .. 30, ^{1}H:0 .. 60, ^{14}N:0 .. 3, ^{16}O:0 .. 4

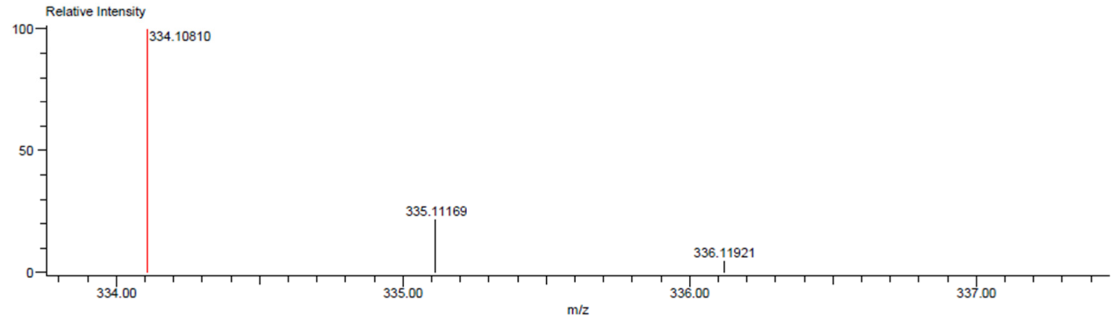

Mass	Intensity	Calc. Mass	Mass Difference (mmu)	Mass Difference (ppm)	Possible Formula	Unsaturation Number
334.10810	1296321.97	334.10793	0.17	0.51	$^{12}C_{20}{}^{1}H_{16}{}^{14}N_{1}{}^{16}O_{4}$	13.5

Figure S29. HRMS spectrum for compound **6**.

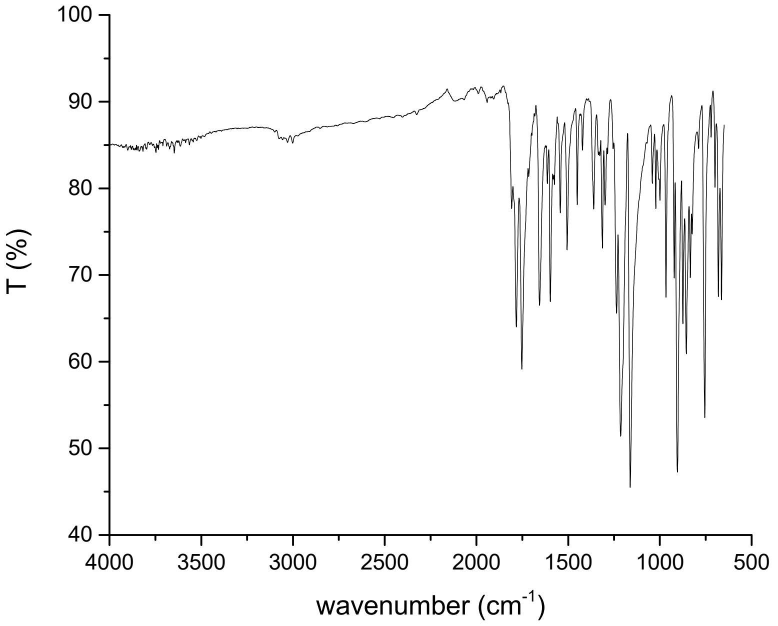

Figure S30. FT-IR (ATR) spectrum for compound **6.**

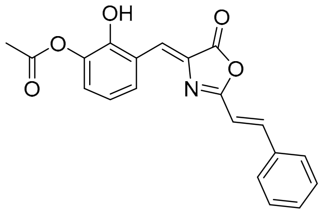

2-hydroxy-3-((Z)-(5-oxo-2-((E)-styryl)oxazol-4(5H)-ylidene)methyl)phenyl acetate (7)

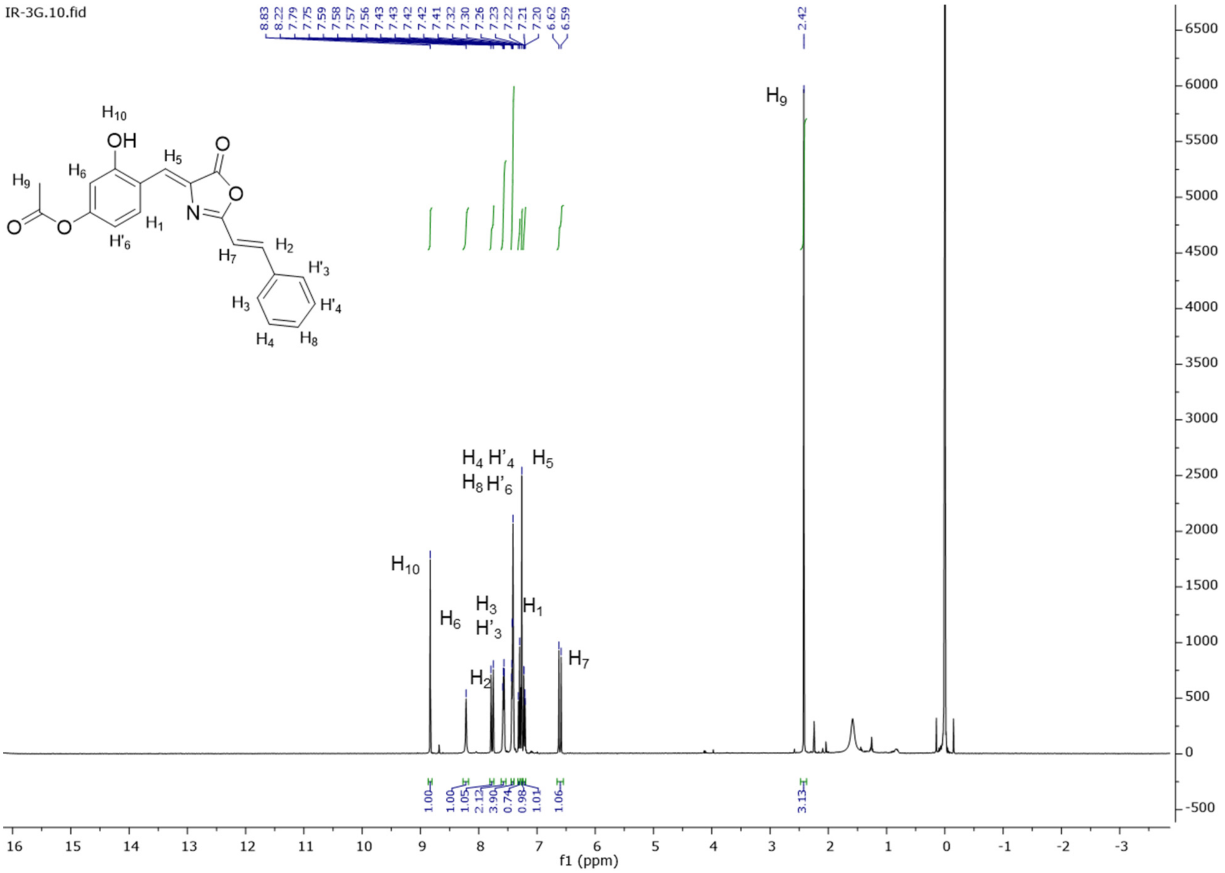

Figure S31. ^{1}H (400 MHz) NMR spectrum for compound **7** in $CDCl_3$ at 298 K.

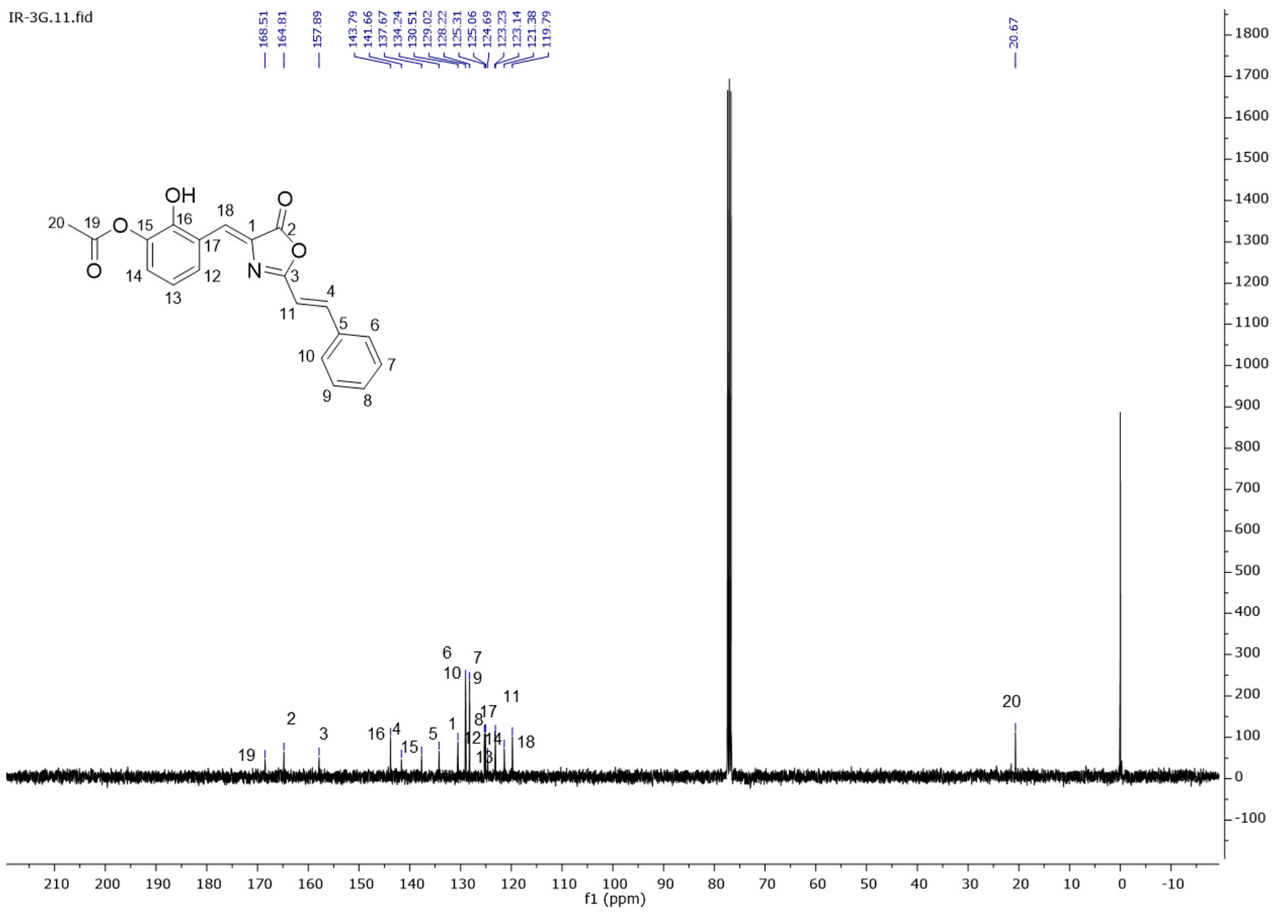

Figure S32. ^{13}C (100 MHz) NMR spectrum for compound **7** at 298 K.

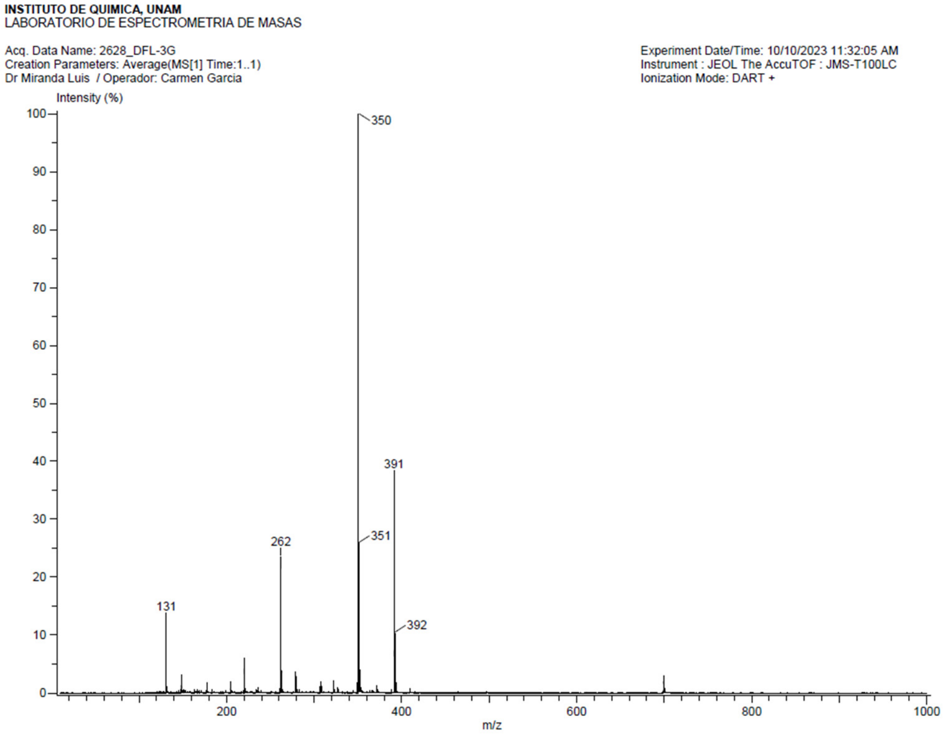

Figure S33. EI-MS spectrum for compound **7**.

Data:2628_DFL-3G
Sample Name:Dr Miranda Luis / Operador: Carmen Garcia
Description:
Ionization Mode:ESI+
History:Determine m/z[Peak Detect[Centroid,30,Area];Correct Base[];Smooth[5]];Correct Base[5.0%];Average(MS[...

Acquired:10/10/2023 11:32:05 AM
Operator:AccuTOF
Mass Calibration data:cal-PEG-600-CG
Created:10/17/2023 7:08:34 PM
Created by:AccuTOF

Charge number:1
Tolerance:3.00(mmu)
Unsaturation Number:0.0 .. 50.0 (Fraction:.5)
Element:^{12}C:0 .. 30, ^{1}H:0 .. 60, ^{14}N:0 .. 3, ^{16}O:2 .. 6

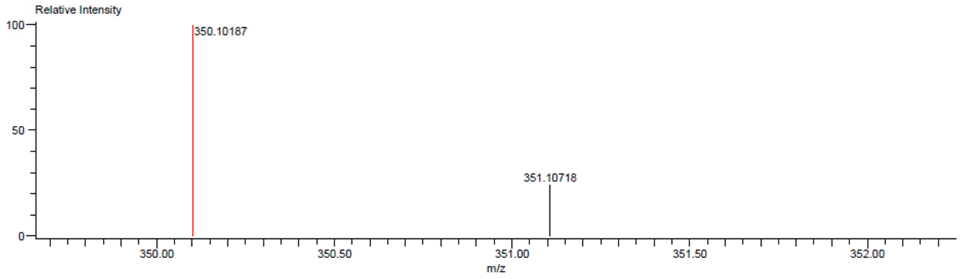

Mass	Intensity	Calc. Mass	Mass Difference (mmu)	Mass Difference (ppm)	Possible Formula	Unsaturation Number
350.10187	66482.60	350.10285	-0.98	-2.79	$^{12}C_{20}{}^{1}H_{16}{}^{14}N_{1}{}^{16}O_{5}$	13.5

Figure S34. HRMS spectrum for compound **7**.

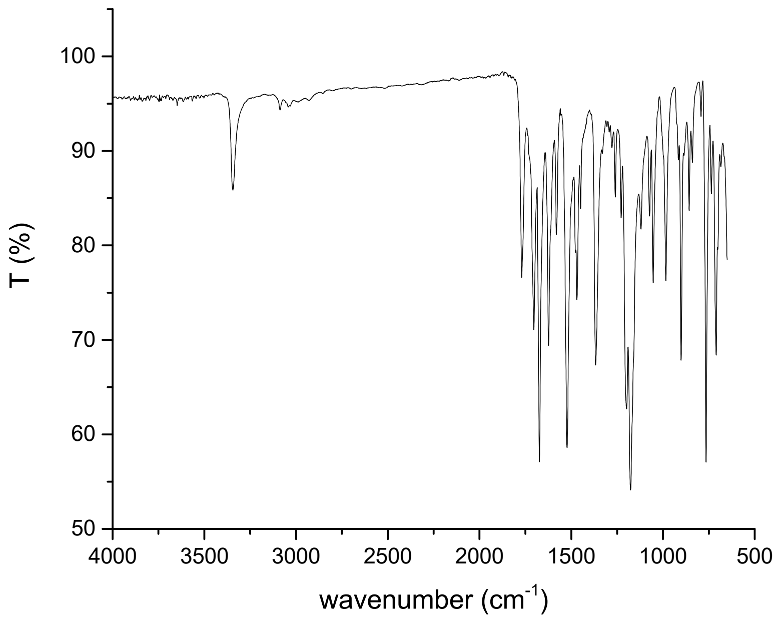

Figure S35. FT-IR (ATR) spectrum for compound **7**.
